# Extracts of Feijoa Inhibit Toll-Like Receptor 2 Signaling and Activate Autophagy Implicating a Role in Dietary Control of IBD

**DOI:** 10.1371/journal.pone.0130910

**Published:** 2015-06-25

**Authors:** Noha Ahmed Nasef, Sunali Mehta, Penny Powell, Gareth Marlow, Tom Wileman, Lynnette R Ferguson

**Affiliations:** 1 Faculty of Medical & Health Sciences, The University of Auckland, Auckland, New Zealand; 2 Biomedical Research Centre, Norwich Medical School, UEA, Norwich, United Kingdom; University of the Pacific, UNITED STATES

## Abstract

**Background:**

Inflammatory bowel disease (IBD) is a heterogeneous chronic inflammatory disease affecting the gut with limited treatment success for its sufferers. This suggests the need for better understanding of the different subtypes of the disease as well as nutritional interventions to compliment current treatments. In this study we assess the ability of a hydrophilic feijoa fraction (F3) to modulate autophagy a process known to regulate inflammation, via TLR2 using IBD cell lines.

**Method:**

Mouse embryonic fibroblasts (MEF) deleted for ATG5, and two intestinal epithelial cells HCT15 and HCT116, were used to test the anti-inflammatory effect of F3 after stimulating the cells with a TLR2 specific ligand PAM3CSK4.

**Results:**

F3 was able to reduce TLR2 specific inflammation and stimulate autophagy in MEFs and HCT15 cells but not in HCT116 cells. The anti-inflammatory effect was reduced in the MEF cells deleted for ATG5. In addition, the activation of autophagy by F3 was enhanced by PAM3CSK4.

**Conclusion:**

F3 of feijoa can interact with cells via a TLR2 specific mechanism and reduce Nuclear factor kappa B (NF-κB) activation in part due to stimulation of autophagy. These results suggest that there is potential benefit in using feijoa extracts as part of dietary interventions to manage IBD in patients.

## Introduction

In healthy individuals, the intestinal immune system has evolved to distinguish between normal gut microbiota and pathogenic bacteria and to respond appropriately to each. The innate immune system protects the body by activating signal transduction pathways via pattern recognition receptors (PRRs) that are expressed on epithelial cells and phagocytic cells. PRRs recognize pathogen-associated molecular patterns (PAMPs) as well as damage associated molecular patterns (DAMPs) [1].

The most widely studied PRRs relevant to inflammatory bowel disease (IBD) are the Toll-like receptors (TLRs). Under normal conditions, PRRs initiate a successful acute inflammatory response resulting in the elimination of the infectious agent followed by resolution of inflammation and tissue repair [2]. During bacterial infection, inflammation is generally beneficial, but if uncontrolled could lead to chronic inflammation. In the case of IBD, this balance is impaired resulting in dysbiosis and chronic inflammation [3]. Furthermore in IBD, prolonged inflammation can lead to increased tissue damage [4], epithelial cell necrosis and the subsequent release of DAMPs [5]. DAMPs have the ability to activate PRRs such as TLR2 [6,7] and in turn induce further secondary inflammation in a repeating cycle that ultimately results in a self-sustaining chronic inflammation [8]. An understanding of the interaction between the microbiota and the host is essential for a better understanding of IBD.

Activation of TLRs can induce autophagy which acts as a defense against bacterial invasion [9,10] and is important in regulating inflammation [11]. Macrophages lacking ATG16L1, an essential autophagy protein, have increased production of the pro-inflammatory cytokine IL1β [12]. Furthermore, macrophages unable to activate autophagy show increased LPS-dependent inflammasome activation suggesting that autophagy regulates production of inflammatory cytokines in these cells [12]. In addition to regulating inflammatory signaling, autophagy may prevent tissue inflammation through its role in clearance of apoptotic cell debris [13] which can prevent secondary necrosis and release of DAMPS that trigger inflammation possibly leading to chronic inflammation.

The primary focus for treating IBD disease is to reduce inflammation or “flare-ups” in the bowel when the disease is active and to keep inflammation at normal levels during time of remission [14]. However, limited success of these treatments accompanied by significant side effects for some patients demonstrates the need for complementary intervention. [15,16]. An obvious way to manage inflammation to improve IBD symptoms includes dietary intervention.

The processes through which dietary extracts interact with the inflammatory and bacterial sensing responses remain unclear. Fruits contain a wide variety of polyphenols that have anti-inflammatory properties and are increasingly regarded as effective protective agents against chronic inflammatory disease [17,18,19,20,21,22,23,24]. Previously we have screened various fruits by fractionating them into five hydrophobic and five hydrophilic fractions to assess for their anti-inflammatory property through TLR2 and TLR4 signaling [25]. From our screen, the third hydrophilic fraction (F3) of feijoa, a South American fruit that is commonly cultivated in New Zealand, was the most efficient in reducing inflammation induced by TLR2 signaling. In this study we have determined whether activation of autophagy by F3 plays a role in reducing TLR2 signaling in mouse embryonic fibroblasts (MEF), HCT15 and HCT116 intestinal cell lines when induced by a synthetic triacylated lipoprotein ligand PAM3CSK4.

## Experimental Section

### Cell line and culture medium

Mouse embryonic fibroblast wild type (MEF) and MEF with autophagy *ATG5* gene knocked out (MEF Atg 5^-/-^) cell lines were provided by Norwich Medical School, University of East Anglia, Norfolk UK. Human colorectal cancer cell lines HCT15 and HCT116 were obtained from the Department of Nutrition, University of Auckland. MEFs and HCT15 cells were maintained in high glucose DMEM containing sodium pyruvate and L-glutamine (Life technologies, USA). HCT116 cells were maintained in RPMI 1640 containing L-glutamine (Life technologies, USA). All media was supplemented with 10% FBS (Life technologies, USA) and 1% penicillin streptomycin (PSG; Life Technologies, USA). Experiments were conducted using DMEM containing sodium pyruvate and L-glutamine (HCT15 and MEFs) or RPMI 1640 (HCT116) (Life technologies, USA) supplemented with 10% FBS at 37°C in a humidified 5% CO_2_ incubator.

### Feijoa Fractionation

Feijoa was fractionated as described previously [25] with the help of Wendy Smith from Plant and Food Research (Ruakura, New Zealand). Briefly, 10 g of ground up freeze dried feijoa was homogenized in 100 ml of ethanol. It was mixed overnight followed by filtering under vacuum. 50 ml of the filtered solution was coated onto 6 g of octadecyl-functionalized silica gel (C18; Sigma Aldrich, USA) and dried using a rotary evaporator. Next, the dried bulk mix was added to a preconditioned separation phase extraction (SPE) cartridge. Two hydrophilic fractions (F1 and F2) were collected from the cartridge by eluting in 100% water. Fractions with decreasing hydrophilic strengths including F3 and F4, F5 and F6, F7 and F8 were collected from the cartridge by eluting in 75% water and 25% ethanol, 50% water and 50% ethanol, 25% water and 75% ethanol respectively. Specifically, F3 was transferred to a speed vacuum concentrator (CentriVap, labconco, USA), for drying overnight at ambient temperature. The dried fraction was then re-dissolved in ethanol and then concentrated using a speed vacuum concentrator (CentriVap, labconco, USA). Each aliquot of F3 contained the equivalent of 1 g of whole food. Each aliquot was sterilized using gamma irradiation at the standard dose of 25–32 Kilogray (Schering-Plough Animal Health Limited, Wellington, New Zealand). The sterilized samples were then dissolved in 1 ml of 20% Dimethyl sulfoxide (DMSO, Sigma-Aldrich, USA) and 80% autoclaved Milli-Q water. Dissolved F3 was then studied using *in vitro* assays.

### Luciferase assay

NF-κB activation was measured under different conditions in the presence and absence of PAM3CSK4, a stimulator of TLR2/1 (called TLR2 from now on) using a luciferase assay. MEF, HCT15 and HCT116 cells were transfected with a pGL4.32[luc2P/NF-κB-RE/Hygro] vector containing NF-κB response elements and a Renilla plasmid luciferase reporter that measured luciferin substrate in a luminometer.

MEFs, HCT15 and HCT116 were seeded into 96-well plates at a cell density of 1x10^5^, 3x10^5^ and 2.5x10^5^ cells/ml respectively. The cells per well were transfected with DNA using 3μl (MEFs) or 2μl (HCT15 and HCT116) of lipofectamine 2000 (Life technologies, USA) and 10μl of 2.3μg/ml pGL4.32[luc2P/NF-κB-RE/Hygro] Vector (Promega, USA):2.2μg/ml Renilla plasmid (Promega, USA) (10:1) dissolved in Opti-MEM media (Life technologies, USA). After 24 hours, the media was removed and the cells were treated with 10mg/ml of feijoa F3, with and without ligand. The control was media in the presence and absence of 100ng/ml PAM3CSK4. The plates were left to incubate at 37˚C for 5 hours, after which the cells were washed and lysed with 20μl of 1x passive lysis buffer (PLB). Using the Luciferase assay kit (Promega, USA), NF-κB expression was measured. 100μl of the luciferase assay reagent was added into each well. Following this, 100μl of the Stop and Glo reagent (Promega, USA) was added to each well in order to measure the constitutive expression of the Renilla plasmid.

The raw sample score (S1 Table) was normalized against the Renilla score to calculate the normalized sample score as follows:

$$x=\frac{\text{Luciferase relative units}\;(\text{RLU})}{\text{Renilla relative units}\;(\text{RLU})}$$ (1)

NF-κB activation ratio was calculated for each sample as follows:

$$x=\frac{\text{Normalized sample with PAM}3\text{CSK}4}{\text{Normalized sample without PAM}3\text{CSK}4}$$ (2)

% NF-κB activation was calculated for each sample as follows:

$$x=\left[\frac{\text{NF-}\kappa \text{B activation ratio of sample}}{\text{NF-}\kappa \text{B activation ratio of media}}\right]*100$$ (3)

% inhibition of NF-κB activation was calculated as follows:

$$x=\left[\frac{\left(\%\text{NF}-\kappa \text{B activation of control}-\%\text{NF}-\kappa \text{B activation of sample}\right)}{\%\text{NF}-\kappa \text{B activation of control}}\right]*100$$ (4)

Each sample was replicated six times and the results were combined, and a mean and standard deviation were calculated.

A student t-test was calculated to measure significance using the holm-sidak method with alpha = 5% in the Graphpad Prism software version 6.01.

### Immunofluorescence and Quantification

MEF, HCT15 and HCT116 cells were grown in 24-well plates on circular 13mm diameter glass coverslips at a cell density of 0.5x10^5^, 3x10^5^ and 2.5x10^5^ cells/ml respectively. The cells were then treated with 10mg/ml of feijoa F3, or controls, with and without ligand. The controls included 1μM Torin1 to activate autophagy, and media. The ligand used was 100ng/ml of PAM3CSK4.

Following 5 hour incubation with fruit extracts and/or PAM3CSK4, the cells were fixed in ice-cold methanol (Merck Millipore, Canada) then washed in PBS. Nonspecific antibody binding was blocked using 2% bovine serum albumin (BSA; Sigma-Aldrich, USA) in PBS for 30 minutes at room temperature. The cells were then immunostained using αLC3B polyclonal primary antibody raised in rabbit (Sigma-Aldrich, USA) and diluted 1:1000 in 2% BSA (Sigma-Aldrich, USA).

Washed cells were incubated in secondary antibody linked to Alexafluor 594 (Abcam, UK). Nuclei were visualized using 4',6-Diamidino-2-Phenylindole, Dihydrochloride (DAPI; Life technologies, USA) and coverslips were mounted using Vectashield hardset (Vector Labs, USA) and sealed with nail polish and visualized using a light microscope.

Quantification of the LC3 puncta indicative of autophagosomes was achieved using Imaris software (Bitplane Software Incorporated, UK). The spot function was used to identify and measure the diameter of fluorescent puncta using the whole z stack for each experiment. An expected spot diameter was defined as 0.4μm. A Gaussian filter was used to remove background noise, smoothing over objects at a distance below 8/9 of the expected spot radius. Local contrast was then used to estimate spot size. The rendered images of the LC3 puncta were colored according to diameter on a graded scale with a diameter of 0.1μm being blue, and anything 1 μm and above being colored red.

### Western Blot

HCT116 cells were seeded at a cell density of 1x10^5^ cells/ml and HCT15 cells at a cell density of 4x10^5^ cells per ml in 2mls of growth media. Cells were incubated for 5 hours with 10mg/ml feijoa F3, 1μM Torin1 and/or 100ng/ml PAM3CSK4. Cells were washed in PBS and extracted using RIPA lysis buffer containing 150mM NaCl, 1% Triton X-100, 0.5% Sodium Deoxycholate, 0.1% SDS, 50mM Tris (pH8) and protease inhibitors cocktail (Promega, USA). The protein was quantified (BCA, Sigma, USA) following the manufacturer instructions.

The proteins were separated by 12% SDS PAGE (Novex Bolt system, Life technologies, USA) run under reducing conditions. Proteins were then transferred to a PVDF membrane, by semi-dry transfer (Bio-rad Trans-blot turbo system, Bio-rad, USA). The membrane was blocked using 5% milk in PBS-Tween for 1 hour at room temperature. LC3 was visualized with the anti-LC3B polyclonal primary antibody raised in rabbit (Sigma-Aldrich, USA), followed by donkey anti-rabbit (CF770 green, mix ‘n’ stain, Sigma-Aldrich, USA). β-actin protein was used as a control for equal lane loading and visualized by incubating with anti-β-actin monoclonal antibody raised in mouse (Sigma-Aldrich, USA), followed by donkey anti-mouse (680R red, mix ‘n’ stain, Sigma-Aldrich, USA).

Fluorescent labeling was visualized using the Odyssey FC (Li-cor, Millenium science, Australia) and semi-quantified using Image Studio Lite version 3.1 with background selected manually.

The ratio for β-actin to LC3 was generated by dividing the band intensity of the media control without PAM3CSK4 by the band intensity of the experimental sample. The generated ratio was multiplied by the LC3B I and II band intensity score to give an adjusted score.

## Results

### Feijoa F3 inhibits TLR2 activation of NF-κB in MEF cells and this may be linked to autophagy

ATG5 is essential for autophagy and MEF ATG5^-/-^ have been used in many studies to determine the role played by autophagy in cellular processes. Control MEF and MEF Atg 5^-/-^ cells were used to test whether autophagy plays a role in regulating the anti-inflammatory effect mediated by feijoa F3.

**Fig 1:**
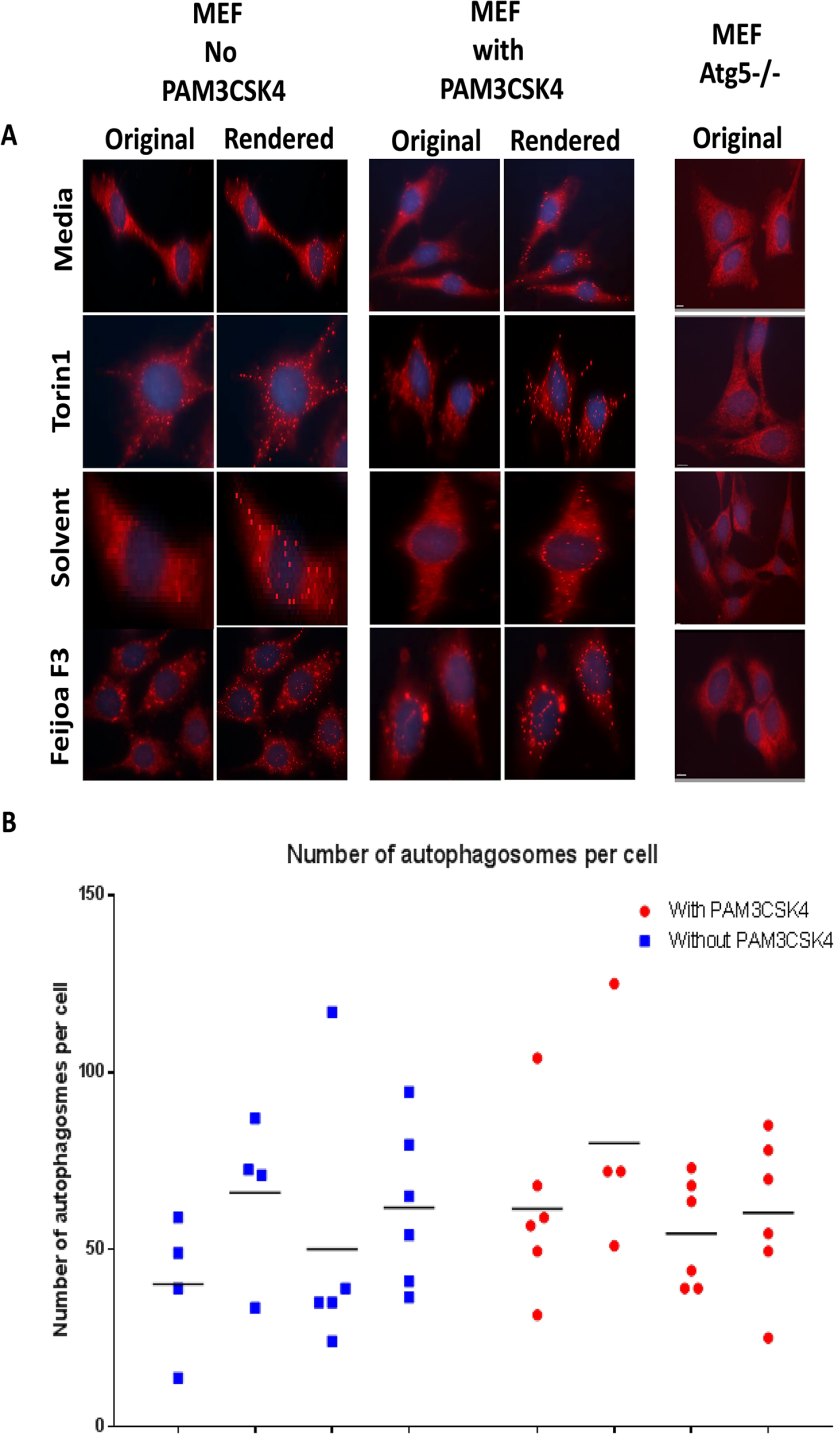
Feijoa F3 activates autophagy in MEF cells. Cells were treated with feijoa F3 at 10mg/ml, Torin1 at 1μM/ml, with and without 100ng/ml PAM3CSK4 stimulation. **A.** Shows the LC3B (marker of autophagy) staining. The original images show puncta in cells that represent autophagosomes. The rendered images are created using the Imaris spot function where the spots in the cells are spheres pseudo colored and represent the autophagosomes. The MEF Atg 5^-/-^ showed no puncta **B.** The puncta were quantified using the spot function in Imaris. Each experimental condition for solvent, media, and feijoa F3 had 3 biological replicates with two technical replicates (giving a maximum of 6 data points) and Torin1 had 2 biological replicates with two technical replicates (giving a maximum of 4 data points). Horizontal line represents median.

Activation of autophagy by feijoa F3 was investigated in MEF and MEF Atg 5^-/-^ cells. The cells were treated with 10 mg/ml of feijoa F3 and an equivalent amount of solvent, 1 μM/ml of Torin1 (a positive marker of autophagy activation) and an equivalent amount of media. Immunofluorescence was used to identify LC3 puncta indicative of autophagosome in the MEF and MEF Atg 5^-/-^ cells in the presence and absence of stimulation by 100ng/ml of PAM3CSK4. LC3 puncta per cell are illustrated in Fig 1A. Puncta were semi quantitated using Imaris software as shown in Fig 1B. Despite the high variability between replicate data points, MEF cells treated with Torin1 showed increased LC3 puncta and numbers of autophagosomes per cell indicative of increased autophagy. Similar results were also observed in the MEF cells treated with feijoa F3 suggesting that the fraction is capable of stimulating autophagy when compared to solvent control and at a level comparable to Torin1 a known stimulator of autophagy.

There was no synergistic effect of PAM3CSK4 with Torin1 or feijoa F3. Fig 1B shows that LC3 puncta were induced in MEF cells by Torin1, PAM3CSK4 and feijoa F3 extract, but these compounds were unable to generate LC3 puncta in MEF Atg 5^-/-^ cells.

**Fig 2:**
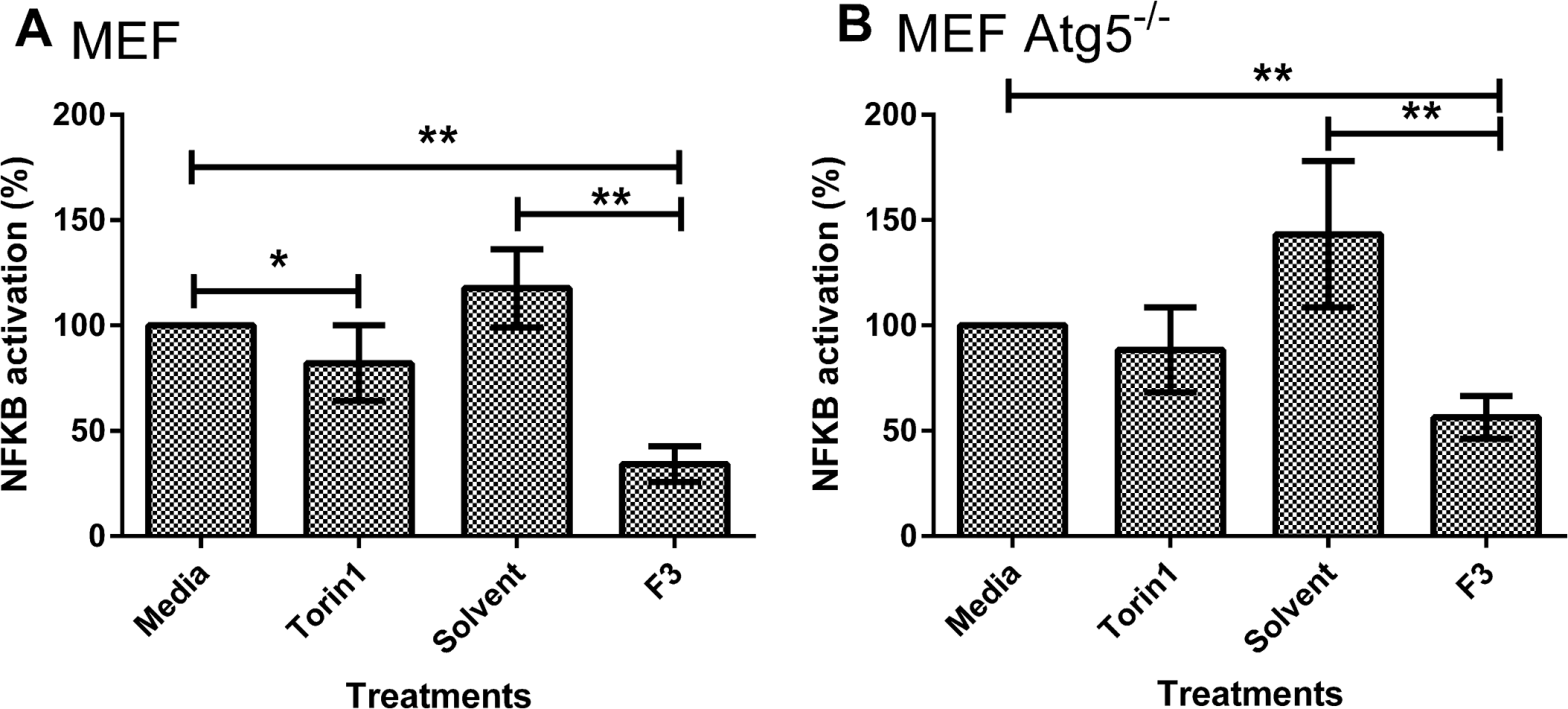
Feijoa F3 inhibits NF-κB activity in A. MEF cells and B. MEF Atg5^-/-^ cells. Cells were treated with feijoa F3 at 10mg/ml, Torin1 at 1μM/ml, with and without 100ng/ml PAM3CSK4 stimulation. **A and B** show the normalized percentage NF-κB activation. Percentage inflammation was calculated using Eq 3. A percentage of greater than 100 shows increased inflammatory response in the presence of the ligand when compared to media (no treatment); and a percentage of less than 100 suggests a decrease in the inflammatory response in the presence of the ligand when compared to media (no treatment) meaning that a condition has inhibited NF-κB signaling. Error bars represent standard deviation (n = 6). ** P-value <0.001 * <0.05.

MEF cells were transfected with a reporter plasmid containing NF-κB promoter-luciferase response cDNA. A luciferase assay was used to measure the activation of NF-κB by PAM3CSK4 as a means of analyzing the inflammatory activity in the cells. Feijoa F3 reduced NF-κB activity by 86.6% (p value <0.001, Fig 2A) compared to the solvent and by 65.8% (p value <0.001, Fig 2A) compared to media in the presence of PAM3CSK4. In MEF ATG5^-/-^ cells, feijoa F3 reduced NF-κB by 86.9% (p value <0.001, Fig 2B) compared to the solvent control and by 45.6% (p value < 0.001, Fig 2B) compared to media. However, when we compared MEF and MEF ATG5^-/-^ cells inhibition of NF-κB by feijoa F3 fell from 70.9% to 58.5% in the absence of ATG5 (p<0.05, Fig 2A and 2B. These results suggest that autophagy contributes to the inhibition of NF-κB production induced by PAM3CSK4 in the presence of feijoa F3. This was supported by the observation that MEF NF-κB activity was reduced by 17.8% (p value <0.05, Fig 2A) in cells treated with Torin1 compared to the media control. MEF ATG5^-/-^ cells did not show reduced activation of NF-κB by Torin1 (Fig 2B), showing that the reduction of NF-κB activation by Torin1 in control cells required ATG5 and therefore activation of autophagy.

These results provide further evidence that feijoa F3 is capable of activating autophagy and that F3 has the potential to inhibit NF-κB activation.

### Feijoa F3 inhibits TLR2 activation of NF-κB in the intestinal cell line HCT15 and activates autophagy

The HCT15 cell line is heterozygous for IBD susceptibility alleles in autophagy and TLR genes [26] making it an ideal cellular model for TLR/autophagy IBD subtype. This includes being heterozygous for the CD14 -260C>T risk allele. CD14 is a receptor that is part of the complex that enhances the sensitivity of LPS to TLR4 [27] and PAM3CSK4 to TLR2/1 [28]. In addition, HCT15 cells were found to be heterozygous for a number of *ATG16L1* IBD risk alleles and has low expression of *ATG16L1* when compared to other intestinal epithelial cell lines (IECs) [26].

**Fig 3:**
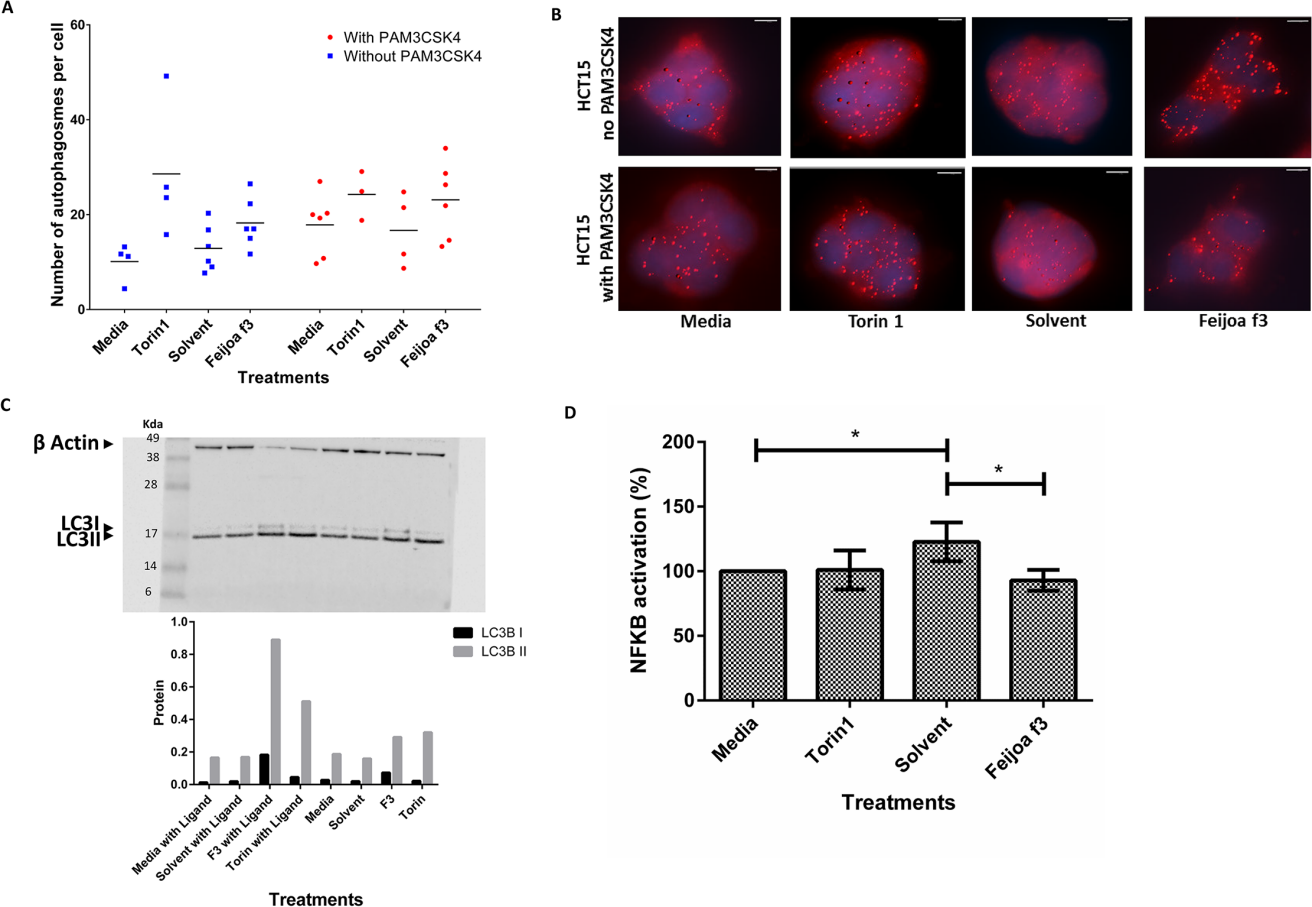
Feijoa F3 activates autophagy and inhibits NF-κB activity in HCT15 cells. Cells were treated with media, Torin1 (1μM/ml), solvent and feijoa F3 (10mg/ml), in the presence and absence of 100ng/ml PAM3CSK4 stimulation. **A and B** show the number of autophagosomes in each cell. The puncta were quantified using the spot function in Imaris. Each experimental condition for solvent, media, and feijoa F3 had 3 biological replicates with two technical replicates (giving a maximum of 6 data points) and Torin1 had 2 biological replicates with two technical replicates (giving a maximum of 4 data points). Horizontal line represents median. **B** is an image representation of **A**. The spots in the cells are the spheres pseudo colored by the Imaris spot function representing the autophagosome puncta that were stained for by LC3B. **C.** shows western blot analysis of autophagy. The Western blot show LC3B I (16kDa) and II (18kDa) protein bands. β-actin (42 kDa) was used as a control. The bottom graph is the semi quantification of the LC3 I and II band intensity scores, adjusted against β-actin band intensity scores. **D** shows the normalized percentage NF-κB activation. Percentage inflammation was calculated using Eq 3. A percentage of greater than 100 shows increased inflammatory response in the presence of the ligand when compared to media (no treatment); and a percentage of less than one suggests a decrease in the inflammatory response in the presence of the ligand when compared to media (no treatment) meaning that a condition has inhibited NF-κB signaling. Error bars represent standard deviation of (n = 6). * P-value <0.05.

Activation of autophagy in HCT15 cells was assessed semi-quantitatively by measurement of LC3 puncta using immunofluorescence. LC3 puncta were not induced in HCT15 cells by solvent alone however, the numbers of LC3 puncta were increased by both Torin1 and feijoa F3 in the presence and absence of PAM3CSK4 compared to media control (Fig 3A and 3B).

Western blot analysis of LC3B was used as a second assay for activation of autophagy (Fig 3C). Addition of lipid to the C-terminus of LC3B I results in formation of LC3II which migrates faster than LC3 I. The band intensities were normalized against β-actin (Fig 3C). Consistent with the results from imaging LC3 puncta, levels of LC3B I and LC3B II remained unchanged after solvent treatment in the presence or absence of PAM3CSK4 stimulation. Torin1 increased the levels of LC3B II in both experiments indicating activation of autophagy. Treatment of HCT15 cells with feijoa F3 increased levels of both LC3B I and LC3B II in the presence and absence of PAM3CSK4 (Fig 3C).

HCT15 cells were transfected with an NF-κB reporter plasmid upstream of a luciferase gene. Addition of solvent to HCT15 cells in the presence of PAM3CSK4 stimulation resulted in an 18.8% (p<0.001) increase in inflammation. Interestingly, despite feijoa F3 being dissolved in solvent, treatment with feijoa F3 decreased basal activation of NF-κB by 25% (P<0.001) as seen in Fig 3D. There was no additional change in inflammation mediated by Torin1 on stimulation with PAM3CSK4 in HCT15 cells (Fig 3D).

### Feijoa F3 has no effect on TLR2/1 activation of NF-κB or autophagy in HCT116 IEC with defective autophagy

HCT116 cells were used as a negative control since these cells contain defective autophagy as a result of the presence of several SNPs in autophagy genes [26]. HCT116 cells were homozygous for two *ATG16L1* IBD susceptibility alleles and two *IRGM* alleles. In addition, HCT116 cells did not express detectable amounts of TLR2 [29]. This cell line was used as a model to test whether feijoa F3 enhanced the cell’s response towards pathogens (mimicked by PAM3CSK4) that the cell is defective in detecting.

**Fig 4:**
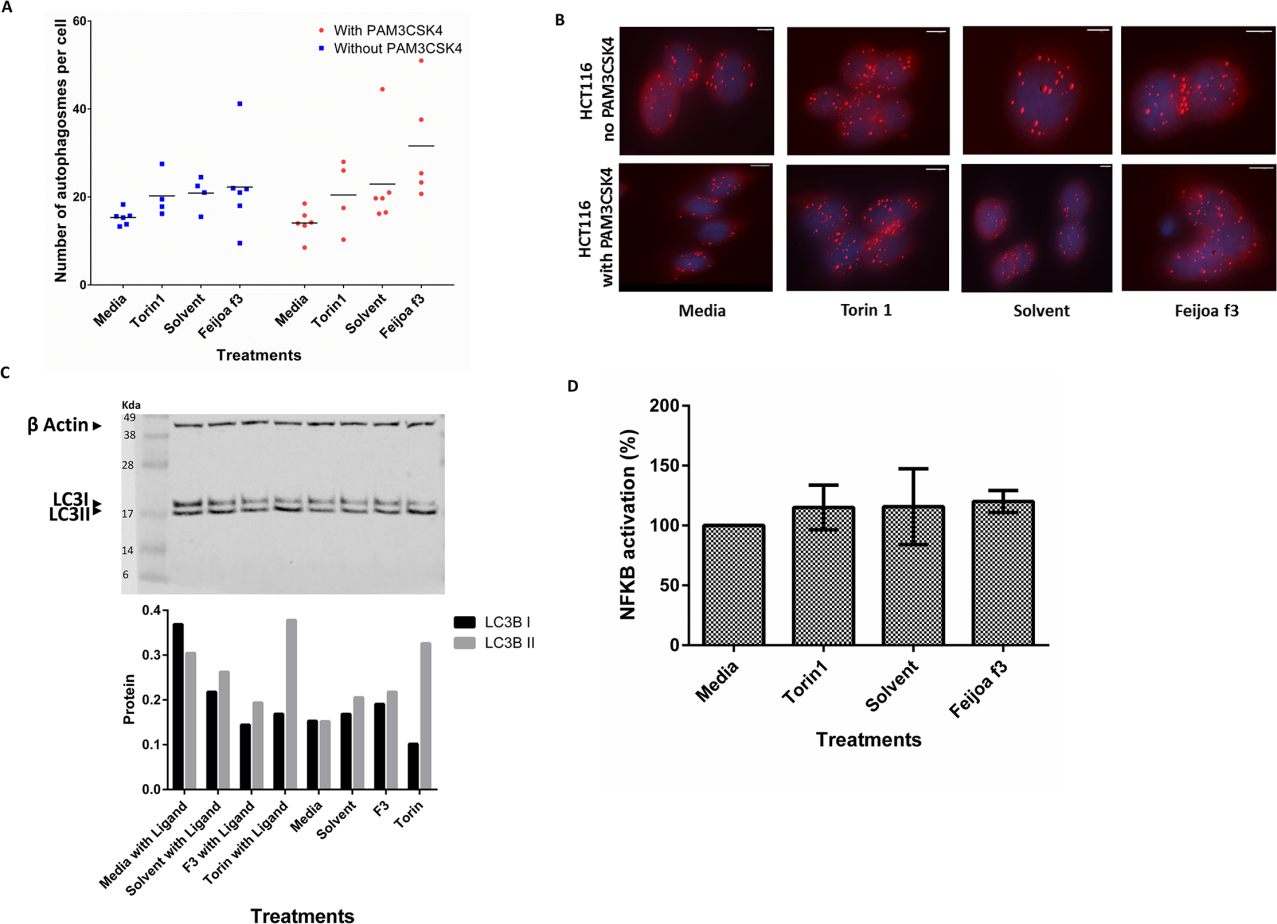
Feijoa F3 does not activate autophagy or inhibit NF-κB activity in autophagy defective HCT116 cells. Cells were treated with media, Torin1 (1μM/ml), solvent and feijoa F3 (10mg/ml), in the presence and absence of 100ng/ml PAM3CSK4 stimulation. **A and B** show the number of autophagosomes in each cell. The puncta were quantified using the spot function in Imaris. Each experimental condition for solvent, media, and feijoa F3 had 3 biological replicates with two technical replicates (giving a maximum of 6 data points) and Torin1 had 2 biological replicates with two technical replicates (giving a maximum of 4 data points). Horizontal line represents median. **B** is an image representation of **A**. The spots in the cells are the spheres pseudo colored by the Imaris spot function representing the autophagosome puncta that were stained for by LC3B. **C.** shows western blot analysis of autophagy. The westerns show LC3B I (16kDa) and II (18kDa) protein bands. β-actin (42 kDa) was used as a control. The bottom graph is the semi quantification of the LC3 I and II band intensity scores, adjusted against β-actin band intensity scores. **D** shows the normalized percentage NF-κB activation. Percentage inflammation was calculated using Eq 3. A percentage of greater than 100 shows increased inflammatory response in the presence of the ligand when compared to media (no treatment); and a percentage of less than 100 suggests a decrease in the inflammatory response in the presence of the ligand when compared to media (no treatment) meaning that a condition has inhibited NF-κB signaling. Error bars represent standard deviation of (n = 6).

To investigate the effect of feijoa F3 on autophagy in HCT116 cells, LC3 puncta formation was measured (Fig 4A and 4B). Interestingly, HCT116 cells treated with solvent alone did not demonstrate an increase in the number of LC3 puncta compared to media control in the absence and presence of PAM3CSK4 stimulation. There was increase in the number of LC3 puncta by both Torin1 and feijoa F3 in presence and absence of PAM3CSK4 stimulation compared to media control (Fig 4A and 4B).

The imaging results were then validated using western blot analysis of LC3B I and LC3B II proteins (Fig 4C). All results were adjusted against β-actin. Consistent with the results from immunofluorescence and imaging, levels of LC3B I and LC3B II remain unchanged after solvent treatment in the presence and absence of PAM3CSK4 stimulation. Similarly, Torin1 increased the levels of LC3B II protein in the presence and absence of PAM3CSK4 stimulation. However, Torin1 did not increase the levels of LC3B I expression. Overall, there appears to be high levels of LC3B I in all the treatments suggesting that there is a defect in the conversion of LC3B I to LC3B II which could be related to the *ATG16L1* SNPs found in this cell line. Cells with media in the presence of PAM3CSK4 had more LC3B I expression compared to LC3B II which was not seen in any of the other treatments. The cells treated with feijoa F3 in the presence of PAM3CSK4 seemed to decrease autophagy stimulation compared to solvent (Fig 4C). This contradicted the immunofluorescent results (Fig 4A and 4B) that show an increase in LC3B when the cells where treated with feijoa F3 in the presence of PAM3CSK4. This may suggest that some of the higher data ranges in the dot plot may be outliers.

HCT116 cells were transfected with a reporter NF-κB reporter plasmid Addition of solvent or feijoa F3 to HCT116 cells in the presence or absence of PAM3CSK4 stimulation did not result in a significant change in NF-κB (Fig 4D). There was no difference in NF-κB activity when HCT116 cells were treated with Torin1 in the presence or absence of stimulation with PAM3CSK4. These results suggest that treatment with feijoa F3 does not enhance the response to TLR2 pathogens and may interact directly with TLR2.

## Discussion

PRRs are found on the surface of intestinal cells and act as sensors of the external environment. In addition, mutations in the PRR pathway are significantly associated with IBD [30,31]. PRRs respond by activating inflammatory signals which result in either an immune response or tolerance.

In our previous study [25] a screen was developed to identify fruit fractions that are capable of inhibiting NF-κB activated via TLR2 as a measure of anti-inflammatory activity. This assay used HEK-Blue engineered cells that can only activate NF-κB through TLR2. Out of the 12 fruits screened, feijoa F3 had the most prominent anti- NF-κB activity and therefore was selected as a good candidate to test further.

To test whether feijoa F3’s anti-inflammatory effects worked through autophagy we tested the difference in the activation of NF-κB inhibition between MEF cells and MEF ATG5^-/-^ cells (cells lacking autophagy). The results suggested that feijoa F3 exhibited inhibition of NF-κB in both MEF and MEF ATG5^-/-^ cell lines. However, NF-κB inhibition was greater in MEFs compared to MEF ATG5^-/-^ cells. These results suggest that autophagy may partly mediate the anti-inflammatory effect exhibited by feijoa F3. This was also supported by the imaging data in Fig 1A and 1B.

Another interesting result from the MEF studies was the significant inhibition of NF-κB activity upon treatment of Torin1 in MEF cells but not the MEF ATG5^-/-^ cells (Fig 2). This may suggest that autophagy is important in the regulation of NF-κB activation. This hypothesis is supported by a study that showed selective degradation of IKK a regulator of NF-κB, using an autophagy mechanism [32]. Further investigation would be required to see whether IKK activation is reduced upon activation of autophagy.

Following this, we used two intestinal cell lines to investigate the interaction of the fruit extract with the intestine. The HCT15 cell line was chosen as it was heterozygous for a number of IBD susceptibility alleles relevant to the biological mechanism tested [26]. HCT116 had defective autophagy and lacked TLR2 expression [26].

Feijoa F3 stimulated autophagy in HCT15 cells based on both the imaging and western results (Fig 3). This suggests that autophagy may be involved in the cell’s interaction with F3. In addition, feijoa F3 dampened the activation of NF-κB that was induced by solvent and primed by PAM3CSK4 in HCT15 cells (Fig 3A). These findings suggest that the compounds in feijoa F3 can enhance the cellular response by dampening the inflammatory response when the machinery is over-sensitive in this cell line.

Contrary to the TLR2 role that has been seen in the HCT15 cells, the results for the role of feijoa F3 on autophagy in HCT116 was inconsistent. However, there appears to be a defect in the conversion of LC3B I to LC3B II in this cell line. In the absence of PAM3CSK4, autophagy was not activated by feijoa F3 based on results from both the imaging or the western results when compared to solvent control. However, in the presence of PAM3CKS4, feijoa F3 may affect autophagy but it is unclear whether it increases or decreases it compared to solvent control. Further studies are required to investigate the effect of feijoa F3 on autophagy in cell lines with defective autophagy.

HCT116 cells showed no effect for feijoa F3 compared to solvent control when the cells were stimulated with PAM3CSK4 (Fig 4D). Since HCT116 does not express detectable amounts of TLR2 according to the literature [29], it is likely that feijoa F3 interacts directly with the TLR2 receptor and it’s machinery to enhance its response to pathogens.

The results from our *in vitro* studies using two intestinal cell lines suggest that in at least a subgroup of patients with an active TLR2 receptor, inflammation can be modulated by activation of autophagy. This study offers an opportunity to develop a personalized approach in identifying and understanding how food interacts with biological processes associated with IBD. Using studies from the literature, we have identified a biological process that is associated with the etiology of the disease in a portion of IBD patients. Feijoa F3 appeared to be dynamic in its interaction with TLR2 and autophagy in the various cell models used. This suggests that the interaction between diet and the host’s immune system is complex and in certain cases, also beneficial. Findings from this study will allow us to investigate how we can use food and compounds isolated from food, to enhance, augment or even complement the body’s interactions with the external environment and in particular towards the microbiota in the gut.

It is important to note the limitations of the study. The results are based on experiments performed on secondary cell lines. The MEF cell lines were generated from mouse fibroblasts and therefore do not necessarily reflect what happens in humans or intestinal cell models. However, the MEF cell line was used as a standard cell line to test autophagy. In addition, NF-κB inhibition was used as an indirect marker of anti-inflammatory effect. However, we did not measure inflammation directly or look at any pro-inflammatory or anti-inflammatory cytokine levels in the cells. Finally, the IECs used are colon cancer cell lines and therefore may not reflect the responses of IECs *in vivo*.

Further studies would be useful to validate the results using primary intestinal cell lines from IBD patients. In particular, it would be useful to investigate the effect of dietary extracts on organoid cultures of intestinal crypts comprising crypt domains surrounding a central lumen and lined by a villus-like epithelium [33]. In addition, measuring inflammation and cytokine levels will give further insight on NF-κB activation or inhibition by feijoa F3.

In this study we have only looked at two aspects of the PRR response, which are inflammation and autophagy. Other aspects of this response should be tested to understand the diet-bacterial sensing process further. This includes looking at the ability of the cell to fight infections with and without feijoa F3.

The gastrointestinal tract is a complex organ that interacts with the external environment to achieve homeostasis. Genetic studies have highlighted that this complex interaction is defective in IBD. The etiology of this disease is still unclear but it is now widely accepted that IBD is a multifactor and heterogeneous disease. For this reason, it is not surprising that the available treatments have limited success in certain individuals. This can also be said for the conflicting results in the association of consumption of healthy food such as fruits and vegetables and the management of IBD as reviewed by Hou *et*. *al*.[34]. Consequently, it can be appreciated that looking at one solution for the disease is not sufficient and that the disease needs to initially be stratified into its various groups in order to get a clearer understanding of how it can be treated.

The central focus of this study was to identify some of the biological mechanisms by which dietary extracts can help IBD patients manage their disease in combination with available medication. This study was motivated by the limited biological understanding of the dietary interactions in IBD. Specifically, we are interested in gaining an increased understanding of the influence of diet on how autophagy interacts with PRRs and the inflammatory response in the context of IBD. We believe that building on this current study and performing others like it, will allow for better understanding of IBD and potentially more effective and targeted interventions for the disease sufferers.

## Conclusion

Our *in vitro* experiments have identified that fraction 3 of feijoa can interact with the bacterial sensing system in various cell models via a TLR2 specific mechanism and activate autophagy to regulate inflammation in the models tested. This project has provided proof of principle that there is potential benefit in using dietary interventions to manage IBD in patients. This can translate into real patient settings to help personalize nutrition based on an individual’s genetic profile.

## Supporting Information

S1 Table Raw sample scores for Renilla (Renilla relative units) and NF-κB (Luciferase relative units). Ligand = PAM3CSK4; F3 = Feijoa Fraction 3.
(DOCX)
