# Supplementary material for: Extracts of Feijoa Inhibit Toll-Like Receptor 2 Signaling and Activate Autophagy Implicating a Role in Dietary Control of IBD
Source: PLoS One. 2015 Jun 25;10(6):e0130910. doi: 10.1371/journal.pone.0130910 (PMC4482396; doi:10.1371/journal.pone.0130910)
Supplement: S1 Table — Ligand = PAM3CSK4; F3 = Feijoa Fraction 3. (DOCX) [file pone.0130910.s001.docx]

S1Table. Raw sample scores for Renilla (Renilla relative units) and NF-κB (Luciferase relative units). Ligand = PAM3CSK4; F3= Feijoa Fraction 3.

| **MEF WT** |  |  |  |  |  |  |  |  |
| --- | --- | --- | --- | --- | --- | --- | --- | --- |
| ***Renilla*** | **Media+Ligand** | **Solvent+Ligand** | **F3+Ligand** | **Torin1+Ligand** | **Media** | **solvent** | **F3** | **Torin1** |
|  | 0.4088 | 0.5281 | 0.4113 | 0.5554 | 0.4439 | 0.5711 | 0.4778 | 0.3868 |
|  | 0.4371 | 0.5420 | 0.3878 | 0.4557 | 0.4048 | 0.4290 | 0.4411 | 0.3617 |
|  | 0.4048 | 0.4323 | 0.4094 | 0.4435 | 0.3751 | 0.4219 | 0.3627 | 0.3441 |
|  | 0.4481 | 0.4300 | 0.3422 | 0.4045 | 0.3249 | 0.3413 | 0.2832 | 0.3784 |
|  | 0.4383 | 0.4782 | 0.4598 | 0.4533 | 0.4848 | 0.3655 | 0.3647 | 0.3845 |
|  | 0.5664 | 0.4473 | 0.5178 | 0.4297 | 0.3941 | 0.3842 | 0.3465 | 0.3964 |
| ***NF-κB*** | **Media+Ligand** | **Solvent+Ligand** | **F3+Ligand** | **Torin1+Ligand** | **Media** | **solvent** | **F3** | **Torin1** |
|  | 157.6 | 165.2 | 42.59 | 81.04 | 28.62 | 28.25 | 25.09 | 15.85 |
|  | 163.1 | 171.8 | 42.97 | 76.78 | 30.95 | 28.00 | 23.99 | 16.17 |
|  | 158.4 | 132.0 | 40.02 | 81.56 | 29.04 | 24.19 | 21.06 | 15.04 |
|  | 143.4 | 168.5 | 41.93 | 67.87 | 26.29 | 21.82 | 19.34 | 15.47 |
|  | 160.6 | 153.6 | 42.05 | 75.03 | 28.98 | 24.68 | 18.38 | 15.44 |
|  | 168.8 | 144.5 | 40.03 | 72.33 | 27.99 | 22.78 | 19.85 | 15.63 |
| **MEF ATG 5^-/-^** |  |  |  |  |  |  |  |  |
| ***Renilla*** | **Media+Ligand** | **Solvent+Ligand** | **F3+Ligand** | **Torin1+Ligand** | **Media** | **solvent** | **F3** | **Torin1** |
|  | 0.2613 | 0.3188 | 0.2676 | 0.2926 | 0.4275 | 0.3003 | 0.3328 | 0.2737 |
|  | 0.3264 | 0.3657 | 0.2404 | 0.2332 | 0.4560 | 0.3008 | 0.2968 | 0.2823 |
|  | 0.3422 | 0.3502 | 0.2659 | 0.2883 | 0.3580 | 0.3029 | 0.3169 | 0.2443 |
|  | 0.2430 | 0.2984 | 0.2492 | 0.2307 | 0.3214 | 0.2977 | 0.3300 | 0.2419 |
|  | 0.2792 | 0.2635 | 0.2335 | 0.2858 | 0.2759 | 0.2777 | 0.5444 | 0.2145 |
|  | 0.3194 | 0.2842 | 0.2532 | 0.2386 | 0.3301 | 0.3420 | 0.3444 | 0.2417 |
| ***NF-κB*** | **Media+Ligand** | **Solvent+Ligand** | **F3+Ligand** | **Torin1+Ligand** | **Media** | **solvent** | **F3** | **Torin1** |
|  | 56.34 | 99.37 | 37.27 | 37.80 | 47.56 | 32.14 | 36.03 | 19.17 |
|  | 103.5 | 96.96 | 36.18 | 37.31 | 47.81 | 31.72 | 34.58 | 18.22 |
|  | 84.52 | 110.6 | 35.78 | 37.41 | 40.40 | 38.15 | 31.99 | 18.60 |
|  | 53.12 | 86.78 | 34.83 | 41.81 | 36.62 | 35.83 | 30.89 | 18.88 |
|  | 83.03 | 85.19 | 30.43 | 38.32 | 31.17 | 29.46 | 40.08 | 17.85 |
|  | 70.24 | 86.90 | 32.18 | 34.90 | 36.02 | 31.60 | 31.76 | 18.74 |
| **HCT15** |  |  |  |  |  |  |  |  |
| ***Renilla*** | **Media+Ligand** | **Solvent+Ligand** | **F3+Ligand** | **Torin1+Ligand** | **Media** | **Solvent** | **F3** | **Torin1** |
|  | 1.281482 | 1.352324 | 0.9924178 | 1.133788 | 1.36327 | 1.266749 | 1.219543 | 1.086582 |
|  | 1.426059 | 1.531814 | 1.252934 | 1.1466 | 1.495753 | 1.361989 | 0.705362 | 1.138593 |
|  | 1.493271 | 1.330193 | 1.004102 | 1.06096 | 1.451253 | 1.697578 | 0.688531 | 1.169456 |
|  | 1.589349 | 1.444055 | 1.100673 | 1.370476 | 1.439101 | 1.479838 | 1.134218 | 1.013841 |
|  | 1.513062 | 1.246887 | 1.21518 | 1.39335 | 1.684941 | 1.49229 | 1.161086 | 1.013875 |
|  | 1.375718 | 1.306655 | 1.32401 | 1.148857 | 1.507441 | 1.446848 | 1.199446 | 1.125131 |
| ***NF-κB*** | **Media+Ligand** | **Solvent+Ligand** | **F3+Ligand** | **Torin1+Ligand** | **Media** | **Solvent** | **F3** | **Torin1** |
|  | 12.64603 | 11.64663 | 8.96973 | 9.333945 | 9.344062 | 7.367191 | 8.287639 | 6.916866 |
|  | 13.12374 | 12.77494 | 9.681145 | 10.96327 | 10.33682 | 8.185777 | 4.236221 | 7.479431 |
|  | 14.15984 | 12.12202 | 8.5249 | 11.43647 | 10.64671 | 8.94245 | 4.570967 | 7.646115 |
|  | 14.58295 | 13.12884 | 9.644203 | 11.48206 | 10.73633 | 8.247108 | 8.141656 | 7.587129 |
|  | 13.63775 | 11.75672 | 10.1346 | 12.00286 | 11.4983 | 8.027672 | 7.879312 | 6.728954 |
|  | 13.42846 | 12.36741 | 9.390663 | 9.981652 | 10.3053 | 7.368412 | 7.665835 | 7.603822 |
| **HCT116** |  |  |  |  |  |  |  |  |
| ***Renilla*** | **Media+Ligand** | **Solvent+Ligand** | **F3+Ligand** | **Torin1+Ligand** | **Media** | **Solvent** | **F3** | **Torin1** |
|  | 17.72 | 11.69 | 15.83 | 16.27 | 12.65 | 14.13 | 12.63 | 13.43 |
|  | 14.99 | 13.89 | 15.63 | 13.19 | 14.00 | 14.65 | 14.19 | 14.00 |
|  | 15.08 | 16.69 | 15.25 | 13.31 | 15.67 | 14.30 | 14.29 | 13.21 |
|  | 14.75 | 16.29 | 16.74 | 13.72 | 13.79 | 14.49 | 17.27 | 14.95 |
|  | 14.41 | 14.82 | 15.86 | 15.10 | 14.52 | 14.89 | 17.65 | 14.93 |
|  | 15.63 | 15.01 | 13.97 | 14.91 | 15.67 | 16.48 | 17.88 | 14.77 |
| ***NF-κB*** | **Media+Ligand** | **Solvent+Ligand** | **F3+Ligand** | **Torin1+Ligand** | **Media** | **Solvent** | **F3** | **Torin1** |
|  | 41.97 | 36.90 | 43.36 | 38.09 | 45.18 | 38.63 | 39.76 | 33.47 |
|  | 43.93 | 37.81 | 46.18 | 37.27 | 47.81 | 42.47 | 42.98 | 39.03 |
|  | 43.50 | 38.42 | 44.81 | 39.26 | 47.25 | 44.15 | 39.20 | 39.41 |
|  | 39.91 | 39.61 | 47.42 | 37.56 | 46.07 | 41.51 | 46.09 | 38.89 |
|  | 39.28 | 41.34 | 43.18 | 37.92 | 45.31 | 40.83 | 45.39 | 40.23 |
|  | 41.93 | 40.35 | 40.28 | 36.16 | 42.33 | 39.83 | 46.51 | 39.78 |
